# Challenges associated with the integration of immuno-oncology agents in clinical practice

**DOI:** 10.1186/s12909-022-03847-0

**Published:** 2022-11-12

**Authors:** Patrice Lazure, Aparna R. Parikh, Neal E. Ready, Marianne J. Davies, Sophie Péloquin, Jeffrey M. Caterino, Robert Lewandowski, Alexander J. Lazar, Suzanne Murray

**Affiliations:** 1 AXDEV Group Inc, 210-8, Place du Commerce, QC J4W 3H2 Brossard, Canada; 2 Department of Medicine, Massachusetts General Hospital Cancer Center, Harvard Medical School, Boston, MA USA; 3 Duke University School of Medicine, Durham, NC USA; 4 Smilow Cancer Center, Yale University School of Nursing, New Haven, CT USA; 5 The Ohio State University College of Medicine, Columbus, OH USA; 6 Northwestern Memorial Hospital, Chicago, IL USA; 7 Departments of Pathology and Genomic Medicine, The University of Texas MD Anderson Cancer Center, TX Houston, USA

**Keywords:** Immuno-oncology, Pharmacodiagnostics, Challenges and barriers, Needs assessment, Continuing medical education, Patient-provider communication

## Abstract

**Background:**

The availability of new immuno-oncology therapeutics markedly impacts oncology clinicians’ treatment decision-making. To effectively support healthcare professionals (HCPs) in their practice, it is important to better understand the challenges and barriers that can accompany the introduction of these agents. This study aimed to establish the types and causes of clinical challenges posed by the introduction of new immuno-oncology agents.

**Methods:**

The mixed-methods design included qualitative in-depth interviews and group discussions with HCPs, in which participants discussed clinical challenges and potential underlying reasons for these challenges. Qualitative findings informed a quantitative survey. This survey investigated the extent and distribution of challenges using HCPs’ self-rating of knowledge, skill, confidence, and exposure to system-level effects. These two phases were conducted sequentially with distinctly stratified samples of oncologists, nurse practitioners (NPs), physician assistants (PAs), pathologists, clinical pharmacists, interventional radiologists, rheumatologists, pulmonologists, and emergency department physicians. Participants were from the United States and had various levels of clinical experience and represented both academic and community-based settings.

**Results:**

The final sample included 107 HCPs in the qualitative phase and 554 in the quantitative phase. Analyses revealed clinical challenges related to the use of pharmacodiagnostics. For example, 47% of pathologists and 42% of oncologists reported skill gaps in identifying the appropriate marker and 46% of oncologists, 61% of PAs, 66% of NPs, 74% of pulmonologists and 81% of clinical pharmacists reported skill gaps in selecting treatment based on test results. Challenges also emerged regarding the integration of immuno-oncology agents, as oncologists, rheumatologists, pulmonologists, clinical pharmacists, PAs, and NPs reported knowledge gaps (74-81%) of the safety profiles of recently approved agents. In addition, 90% of clinical pharmacists reported skill gaps weighing the risks and benefits of treating patients with immuno-oncology agents while affected by lupus. Finally, patient communication challenges were identified: HCPs reported difficulties discussing essential aspects of immunotherapy to patients as well as how they might compare to other types of therapies.

**Conclusion:**

The challenges highlighted in this study reveal substantial educational gaps related to the integration of immuno-oncology agents into practice for various groups of HCPs. These findings provide a strong base of evidence for future educational initiatives.

**Supplementary Information:**

The online version contains supplementary material available at 10.1186/s12909-022-03847-0.

## Background

The evolving understanding of the complex interplays of both tumor and host immune responses, including the tumor microenvironment, has led to a deeper understanding of how cancer evades the immune system [1] and contributed to the development of novel treatment options such as immuno-oncology (IO) agents. These options, which include monoclonal antibodies (mAbs), checkpoint inhibitors, and T-cell therapies, have hastened the growth of pharmacodiagnostics to aid treatment selection for patients, although, these options do require careful monitoring of patients’ treatment responses and immune-mediated treatment toxicities by healthcare providers (HCPs) [2–4].

Existing literature suggests the integration of IO agents and the use of pharmacodiagnostics to support their use in practice poses many challenges, including the identification of clinically valuable biomarkers [5] and the management of adverse side effects for patients [6]. However, little is known about HCPs’ knowledge, skills, confidence, and attitudes related to pharmacodiagnostics, the integration of IO agents into clinical practice, and the management of IO-related adverse events (AEs). This study aims to identify and document the challenges and barriers (at the individual, team, and systems levels) currently experienced by a diversity of HCPs integrating new immunotherapy agents in their practice or being directly impacted by their integration. The goal of this study was to generate evidence that can contribute to informing continuing medical education, continuing professional development and other types of interventions to support personalized care for cancer patients.

## Methods

This study utilized a mixed-methods sequential design [7] consisting of a qualitative exploratory phase followed by a quantitative validation phase. The targeted sample included oncologists, nurse practitioners (NPs), physician assistants (PAs) specialized in oncology, pathologists, clinical pharmacists, interventional radiologists, rheumatologists, pulmonologists, and emergency department (ED) [8] physicians located in the United States with at least three years of practice. ED physicians were included in the study, as they are frequently the first physicians who evaluate patients with symptoms of potential immune toxicity given such patients have high rates of ED use and early identification is critical to their care [9–12]. Rheumatologists were included considering immune checkpoint inhibitors may cause inflammatory symptoms of significant importance to them [13], creating new educational needs for this audience of learners[14]. Pulmonologists were included in the study as well, since at the time of data collection, the field of immunotherapy was considered to be particularly fast evolving with respect to lung cancer [15, 16]. To be included in the study, a minimum yearly caseload of patients considered for and/or ongoing immunotherapy was required: 20 for the core oncology team (i.e., oncologists, NPs, PAs); 5 for clinical pharmacists, interventional radiologists, rheumatologists, and pulmonologists; 2 for ED physicians. Pathologists were required to have a minimum of 10 samples per year analyzed to inform the diagnostic or treatment of patients being considered for immunotherapy treatment.

For each phase of data collection, participants were recruited from two distinct online panels (one for qualitative phase, one for quantitative phase), which databases contained healthcare professionals potentially meeting base inclusion criteria (profession/specialty, US-located practice) and who agreed to be contacted for research participation when studies are available in their field. Both panels operated in compliance with the ICC/ESOMAR code of conduct and ethical standards for market and social research [17]. Invitations were sent electronically and included a link to a secure website directing participants to a screening questionnaire and an informed consent form. For both phases, participants were purposively sampled by profession/specialty, practice setting (i.e., academic vs. community-based), and years of practice (3–10 years, 11–20 years, 21 years or more) to ensure a variety of perspectives were included. Data was also collected on participant’s work location (urban /suburban /rural), state, and caseload of patients currently or considered for treatment with immuno-therapy.

This study received approval from Veritas IRB, an international independent ethical review board. Informed consent was obtained from each participant and participants were advised of the practices to maintain confidentiality of their data and the anonymization of data in dissemination. Participants received IRB-approved compensation based on their profession/specialty and the nature of their participation whether interview ($225–300 USD), discussion group ($300–400 USD), or survey ($40–76 USD) in accordance with best practices in research [18].

The first phase of data collection consisted of individual 45-minute semi-structured telephone interviews and 90-minute virtual group discussions with HCPs facilitated by trained interviewers. The guide used for the interviews and group discussions (see Supplementary Material A) drew from challenges identified in the literature and discussions between a panel of both clinical and educational experts. The interviewers probed for both emergent challenges, in terms of individual knowledge, skills, confidence, and attitudes, as well as challenges and barriers experienced within their own interprofessional team, clinical practice setting, and healthcare systems.

Data analysis and coding of qualitative data were based on a thematic analysis approach [19] and directed content analysis [20] using NVivo (QSR International Pty Ltd, Version 12, 2018). Analysis consisted of categorizing and organizing text data into overarching themes and topics included in the interview guide using a coding matrix. This matrix was revised and modified according to emerging themes throughout the process. This was followed by a multi-disciplinary interpretation of findings among co-authors and members of the research team.

The second phase of data collection employed a 20-minute online survey to validate the representativeness of qualitative findings across professions/specialties, practice settings, and years of practice. To maximise relevance, individual survey items varied according to the participant’s professional role (see supplementary material B). Participants were asked to rate their current level of knowledge and skill according to professional expectations using a five-point Likert-type scale (1 = no knowledge/skill to 5 = expert knowledge/skill). Participants were also asked to rate their perceived level of confidence performing a task via a 101-point visual analogue scale (0 = no confidence to 100 = high confidence). Participants’ attitudes towards and experience of interprofessional, contextual, and systemic barriers were assessed by asking their level of agreement (1 = strongly disagree to 5 = strongly agree) with opinion statements. For each item, participants were provided with the opportunity to select “not relevant to my current practice.”

Survey knowledge and skill items were re-categorized as low levels of knowledge/skill (1-3) vs. high levels of knowledge/skill (4-5). The agreement ratings from the 5-point Likert scale were regrouped in 3 categories: “disagree or strongly disagree,” “neither agree nor disagree,” “agree or strongly agree.” For each item, participants who answered “not relevant to my current practice” were excluded from analysis. Cross-tabulations and chi-squares tests were then run to assess differences in knowledge, skill, and agreement across subgroups (i.e., professions/specialties, practice settings, and years of practice). Kruskal Wallis H and ANOVA tests were run to assess differences in confidence ratings across identical subgroups. All statistical analyses were performed using SPSS 26.0 software (IBM Corporation, Armonk, NY, 2019), with an α = 0.05, and comparisons are presented as significantly different when *p* < .05.

Findings from the qualitative and quantitative phases of the study were triangulated [21] with the current literature and expertise of co-authors used to contextualize, and examine data from a multi-faceted perspective, and to identify areas of greatest need for future interventions. Triangulation involved a process of identifying areas where quantitative data can help determine the extent and distribution of phenomena identified in the qualitative phase and informed by literature. These data sources and findings were interpreted and contextualised for clinical relevance by clinical experts (co-authors ARP, NER, MJD, JMC, RL, and AJL).

## Results

A total of 661 HCPs participated in the study: 107 in the qualitative phase and 554 in the quantitative phase (Table 1). In terms of years of practice, 40% of participants had 3–10 years of  practice, 40% had 11–20 years, and 20% had 21 years or more. Sample distribution by years of practice varied by profession/specialty. Most participants practiced in academic settings. For the qualitative phase, 2375 potential participants were contacted, of whom 163 completed the online screener (response rate = 7%) and 107 were eligible and participated. For the quantitative phase, 3000 potential participants were contacted, 1359 completed the screener (response rate = 45%), 587 were eligible and 554 participated (eligibility rate of screened participants = 41%; completion rate of eligible participants = 94%).

**Table 1:** Sample description by phase (qualitative and quantitative), profession/specialty, years of practice and setting

Profession/ Specialty	Oncologist (*n* = 105)	Rheumatologist (*n* = 52)	Emergency department physician (*n* = 52)	Pulmonologist (*n* = 52)	Pathologist (*n* = 52)	Interventional radiologist (*n* = 53)	Clinical pharmacist (*n* = 107)	Physician assistant *(n* = 83)	Nurse practitioner (*n* = 105)	Total (*n* = 661)
**Study component**										
Interviews	17	8	8	8	8	8	8	9	8	82
Discussion groups	---	---	---	---	---	---	9	7	9	25
Surveys	88	44	44	44	44	45	90	67	88	554
**Years of practice**										
3–10 years	43	12	15	19	23	25	41	50	37	265
11–20 years	43	25	21	19	19	21	43	27	47	265
21 + years	19	15	16	14	10	7	23	6	21	131
**Setting**										
Academic hospital or medical center	36	10	17	18	23	24	38	29	36	231
Community clinic	0	2	0	2	0	1	6	4	5	20
Government medicine	1	0	0	2	2	1	2	0	0	8
Hospital (Community)	11	3	18	2	11	10	42	10	8	115
Multi-specialty physician group practice	13	17	2	10	6	7	7	15	31	108
NCCN/NCI cancer center	1	0	0	0	0	0	5	1	3	10
Single-specialty physician group practice	36	11	15	14	9	9	1	20	20	135
Solo practice	7	9	0	4	0	1	1	4	1	27
Other	0	0	0	0	1	0	5	0	1	7

Challenges were identified under the following three themes: (1) selection of pharmacodiagnostics (e.g., PDL1, tumor mutation burden tests, genomic testing) based on knowledge of actionable biomarkers and therapies, (2) integration of new immuno-oncology agents considering their efficacy and safety for specific patient profiles (e.g., tumor type, comorbidities), and (3) communication with patients about treatment preferences.

### Selection of pharmacodiagnostics based on knowledge of actionable biomarkers and therapies

During interviews and discussion groups, pathologists mentioned that they often received an insufficient amount of tumor sample to complete the multitude of requested molecular tests. A greater proportion of oncologists (75%) than pathologists (44%) agreed with the statement: “I expect other HCPs in charge of completing the biopsy to know exactly how much tissue is required” (Table 2).

**Table 2:** Percentages of participants agreeing / disagreeing with different statements, by profession/specialty

		% (n) agree or disagree with statement^a^									
**Statement**		Oncologist	Rheuma-tologist	Emergency Department Physician	Pulmo- nologist	Path- ologist	Interventional radiologist	Nurse practitioner	Physician assistant	Clinical Pharmacist	Asymptotic Significance (2-sided)^b^
*“In my setting, electronic medical records are not always up to date regarding new treatments”(n=547)*	Agree	49.4% (43)	62.8% (27)	48.8% (21)	65.9% (29)	61.9% (26)	48.8% (21)	31.8% (28)	44.8% (30)	44.4% (40)	0.015
	Neutral	23% (20)	14% (6)	20.9% (9)	13.6% (6)	7.1% (3)	25.6% (11)	19.3% (17)	19.4% (13)	23.3% (21)	
	Disagree	27.6% (24)	23.3% (10)	30.2% (13)	20.5% (9)	31% (13)	25.6% (11)	48.9% (43)	35.8% (24)	32.2% (29)	
*“Despite the volume of new agents available, very few constitute real innovations” (n=375)*	Agree	43.2% (38)	N/A	N/A	N/A	N/A	43.2% (19)	23.9% (21)	32.8% (22)	35.2% (31)	0.016
	Neutral	33% (29)	N/A	N/A	N/A	N/A	34.1% (15)	29.5% (26)	26.9% (18)	38.6% (34)	
	Disagree	23.9% (21)	N/A	N/A	N/A	N/A	22.7% (10)	46.6% (41)	40.3% (27)	26.1% (23)	
*Current guidelines do not reflect current treatment landscape (n=374)*	Agree	42.0% (37)	N/A	N/A	N/A	N/A	59.5% (25)	21.6% (19)	37.9% (25)	32.2% (29)	0.001
	Neutral	39.8% (35)	N/A	N/A	N/A	N/A	31.0% (13)	39.8% (35)	27.3% (18)	44.4% (40)	
	Disagree	18.2% (16)	N/A	N/A	N/A	N/A	9.5% (4)	38.6% (34)	34.8% (23)	23.3% (31)	
*“I prefer prescribing treatments I am familiar with, unless the risk-reward is really important” (n=275)*	Agree	64.4% (56)	N/A	43.2% (19)	N/A	N/A	78.1% (32)	49.4% (41)	57.8% (37)	N/A	0.011
	Neutral	24.1% (21)	N/A	34.1% (15)	N/A	N/A	19.5% (8)	24.1% (20)	20.3% (13)	N/A	
	Disagree	11.5% (10)	N/A	22.7% (10)	N/A	N/A	2.4% (1)	26.5% (22)	21.9% (14)	N/A	
*“I expect other health care professionals in charge of completing the biopsy to know exactly how much tissue is required” (n=247)*	Agree	74.4% (32)	N/A	N/A	64.3% (27)	44.2% (19)	N/A	76.2% (32)	67.4% (29)	82.4% (28)	0.000
	Neutral	23.3% (10)	N/A	N/A	16.7% (7)	16.3% (7)	N/A	11.9% (5)	23.3% (10)	8.8% (3)	
	Disagree	2.3% (1)	N/A	N/A	19.0% (8)	39.5% (17)	N/A	11.9% (5)	9.3% (4)	8.8% (3)	

N/A = Question was not asked to this profession/specialty sub-group

^a^Participants indicated their level of agreement to these statements using a 5-point scale: 1-Strongly agree, 2- Agree, 3- Neither agree nor disagree, 4- Disagree, 5- Strongly disagree. This table has grouped responses into Agree (1, 2) Neutral (3) and Disagree (4, 5)

^b^Significance across all profession/specialty sub-groups determined by two-sided Pearson’s chi-square tests

*“The biggest challenge I have is streamlining of the work and knowing which specific markers will be ordered on a specific patient and then the second step, not only which markers they* [oncologists] *want to test it but which methodologies they would like to use.“*

-Pathologist, Colorado.

Nearly half of pathologists (47%) and oncologists (42%) self-reported low skill levels identifying markers that characterize the progression of a specific type of cancer (Table 3). In addition, 46% of oncologists, 61% of PAs, 66% of NPs, 74% of pulmonologists and 81% of clinical pharmacists reported low skill levels identifying viable treatment options based on pharmacodiagnostics test reports (Table 3).

“Those are the things we use to try to define select patients, but the challenge is, every day, I hear there’s a new definition of what positive means.“

-Oncologist, New York.

### Integration of new immuno-oncology agents considering their efficacy and safety for specific patient profiles

Over three-quarters of surveyed oncologists, rheumatologists, pulmonologists, clinical pharmacists, PAs, and NPs reported low knowledge levels of the safety profile for Avelumab (81%), Alemtuzumab (78%), and Belimumab (77%) [Table 3]. Of the seven other IO agents for which the knowledge of safety profile was asked, low knowledge levels were reported by a proportion of respondents varying between 42% and 74% (Durvalumab (74%), Atezolizumab (71%), Ipilimumab (68%), Pembrolizumab (62%), Nivolumab (59%), Infliximab (58%) and Rituximab (42%). Barriers to staying current with new and emerging IO agents included time and resources required to update electronic medical systems upon approval of pharmaceutical and therapeutics administration teams.

**Table 3:** Percentages of participants reporting low levels of knowledge/skill by profession/specialty

**Knowledge or skill**	**Item**	**Profession/Specialty**									**Sig.**^**a**^
		Onco-logist	Rheuma-tologist	Emergency department physician	Pulmo-nologist	Patho-logist	Interventional radiologist	Clinical pharmacist	Physician assistant	Nurse practitioner	
Knowledge	Safety profile of Avelumab	70.5% (62)	90.5% (38)	N/A	97.7% (42)	N/A	N/A	82.2% (74)	88.1% (59)	72.9% (62)	0.000
	Safety profile of Alemtuzumab	64.4% (56)	85.7% (36)	N/A	95.2% (40)	N/A	N/A	80.0% (72)	86.6% (58)	70.9% (61)	0.000
	Safety profile of Belimumab	79.3% (69)	15.9% (7)	N/A	95.3% (41)	N/A	N/A	84.4% (76)	88.1% (59)	82.1% (69)	0.000
	Best practices for treatment of patients with lupus using immuno-oncology agents	79.8% (63)	6.8% (3)	N/A	N/A	N/A	97.4% (38)	93.2% (82)	83.1% (54)	82.1% (69)	0.000
	Best practices for treatment of patients with rheumatoid arthritis or ankylosing spondylitis using immuno-oncology agents	76.9% (60)	6.8% (3)	N/A	N/A	N/A	87.5% (35)	82% (73)	83.1% (54)	69.1% (58)	0.000
	Best practices for treatment of patients with psoriasis and/or psoriatic arthritis using immuno-oncology agents	71.8% (56)	4.6% (2)	N/A	N/A	N/A	89.7% (35)	85.1% (74)	82.8% (53)	68.2% (58)	0.000
	Sources of information that can improve patients’ understanding of immuno-oncology agents	64.8% (57)	65.9% (29)	92.7% (38)	73.2% (30)	N/A	75% (33)	75.3% (67)	59.7% (40)	43.9% (38)	0.000
	Sources of information that can mislead patients’ understanding of immuno-oncology agents	67% (59)	75% (33)	95.1% (39)	80.5% (33)	N/A	83.7% (36)	80.9% (72)	64.2% (43)	56.8% (50)	0.000
Skills	Explaining to patients the difference between immuno-oncology agents and chemotherapy	37.5% (33)	45.2% (19)	82.5% (33)	N/A	N/A	57.1% (24)	65.1% (56)	33.3% (22)	19.3% (17)	0.000
	Weighing the risks and benefits of treating patients with lupus with immuno-oncology agents	74.1% (60)	6.8% (3)	N/A	N/A	N/A	94.4% (34)	89.5% (77)	76.6% (49)	76.5% (62)	0.000
	Identifying viable treatment options based on pharmacodiagnostic test reports	45.5% (20)	N/A	N/A	73.7% (28)	N/A	N/A	81% (34)	60.5% (26)	65.9% (29)	0.008
	Identifying markers that will characterize the progression of a specific type of cancer	41.9% (18)	N/A	N/A	72.5% (29)	46.5% (20)	81.4% (35)	76.2% (32)	59.1% (26)	54.5% (24)	0.000

N/A = Question was not asked to this profession/specialty sub-group

^a^Significance across all profession/specialty sub-groups determined by two-sided Pearson’s chi-square tests

*We are dependent on our IT pharmacy team to get that information into* [EHR System]. *So it’s a rate-limiting step in ensuring that the system is safe, especially as we look at new agents.*

-Clinical pharmacist, Ohio

An average of 48% of surveyed HCPs agreed that electronic medical records were not always up to date regarding new treatments (Table 2).

During the qualitative phase, HCPs expressed uncertainty regarding the value of integrating new and emerging IO agents due to a lack of clinical evidence comparing the safety and efficacy of agents.

*Does it work? What kind of improved clinical benefit will it provide? Meaning, that if I already have thought of Y for lung cancer* […] *Now, your product Z coming out looks exactly the same.*

-Oncologist, New York

Almost half (43%) of oncologists surveyed agreed with the statement “despite the volume of new agents available, very few constitute real innovations” (Table 2). Further, an average of 60% of HCPs (including 64% of oncologists) agreed with the statement: “I prefer prescribing treatments I am familiar with, unless the risk-reward is really important” (Table 2).

Comorbidities compounded reluctance to pursue treatment with IO agents, particularly if immune-mediated.

You always wonder in those patients if you’re at risk of making those things worse by giving them these drugs, but you don’t necessarily want to refrain from giving these people, potentially, treatment that could help them.

-PA, Colorado.

Low knowledge levels of best practices when using IO agents for patients with comorbidities were prominent in cases of lupus, rheumatoid arthritis or ankylosing spondylitis, and psoriasis and/or psoriatic arthritis (Table 3). 74% of oncologists reported low skill levels weighing the risks and benefits of treating patients with IO agents when affected by lupus (Table 3). There was a significant difference in the skill level of HCPs when weighing the risks and benefits of IO treatment for patients with lupus according to the participant’s years of practice: 81% of HCPs practicing 3–10 years reported low skill levels compared to 71% for 11–20 years and 57% for 21 + years (*p* < .001).

### Communicating with patients about their treatment preferences

Challenges at the individual, interprofessional, and system levels were identified when communicating essential aspects of immunotherapy to patients and how immunotherapy compares to other types of therapies, including setting expectations about treatment and how progression is measured.

Participants in the qualitative phase shared their perspectives regarding the patient’s understanding of immunotherapy, which they believe to be dominated by assumptions about chemotherapy.

*There are patients that still don’t quite understand what they’re on* […] *they will say that they’re on chemotherapy, not quite understanding that even if it’s only immunotherapy they’re on, they still call it chemotherapy.*

-NP, Texas.

Over 65% of all HCPs in the cohort reported low knowledge levels of information sources relevant to patients’ understanding of IO agents, including those that might be misleading patients (Table 3). 45% (45%) of all HCPs surveyed reported low skill level in explaining to patients the difference between immunotherapy and chemotherapy, a challenge reported in greatest frequency among ED physicians (82%) (Table 3).

Median confidence levels of all HCPs in the study were lower (< 75%) in the following areas of patient-provider communication: setting patient expectations about immunotherapy (median = 73; IQR = 35) and explaining to patients their ineligibility for immunotherapy (median = 70; IQR = 39), and/or why they are not responding positively to a given IO agent (median = 69; IQR = 39) (Table 4). Differences in median confidence levels were found between professions/specialties with regards to all three items (Table 4).

**Table 4:** Median confidence levels reported by profession/specialty engaging in key patient-provider communication tasks

	Median confidence level (Interquartile range) when:		
**Profession/** **Specialty**	*Setting realistic expectations with my patients regarding the impact of immuno-therapy on their cancer*	*Explaining to my patient their ineligibility for immuno-therapy, due to health status*	*Explaining to my patient why they are not responding positively to a given immuno-therapy agent*
**Oncologist**	76 (28)	72 (31)	70 (30)
**Interventional radiologist**	50 (39)	47 (53)	55 (59)
**Nurse Practitioner**	80 (27)	80 (31)	75 (37)
**Physician Assistant**	78 (27)	79 (24)	78 (27)
**Clinical Pharmacist**	56 (38)	50 (46)	51 (48)
Asymptotic Significance	*p* < .001	*p* < .001	*p* < .001
Kruskal-Wallis H	**53.686**	**40.371**	**57.365**

Multiple barriers to providing patient education were reported in the qualitative phase including patients’ state of mind when burdened with stress, the volume of important detailed information to communicate, and patients’ declining health status. These barriers cumulatively impact the knowledge, skills, and confidence needed to establish and maintain rapport with the patient.

There’s also a psychological component to some of these patients…they’re frustrated and challenged mentally and socially, and medically as well as to what to do if this therapy isn’t working, and that’s a challenging discussion to have with a patient.

-ED physician, South Carolina

HCPs expressed a few ways that might help them better address communication with patients. These included easy-to-understand patient education resources on how to comprehend the progress of their treatment and a clear process to define who is responsible for patient education, both within and outside the clinical setting.

## Discussion

The results of this mixed-methods study led to the identification of several shared challenges experienced by a variety of HCPs involved in the care of patients with cancer in the United States. More specifically, the findings illustrate the burden on HCPs caused by increased clinical complexity resulting from the recent integration of IO agents as options to treat different types of cancers.

At the time of this study, IO therapy could be considered a relatively new treatment approach. It can be hypothesized that the findings of this study, specifically the lack of knowledge about the new agents, their safety profiles, and best practices in caring for patients with comorbidities may impact the confidence of HCPs to integrate new IO agents into practice. In 2020, Helmberger reported on the limited availability of data on the risks associated with IO agents and rapid development of this field [22]. Our results showed uncertainty regarding safety and the value of IO agents and a conservative approach to using newer IO therapies. This highlights the need to further develop continuing medical education (CME) and continuing health education [23] that provides clinicians with opportunities to discuss currently available assets, such as the often updated treatment guidelines (such as those published by NCCN), emerging safety data of IO agents, and provide the chance to exchange with peers who have experience working safely with these molecules.

Current literature on the potential of IO therapy has addressed - to some extent - the rapid evolution and ensuing challenges for practice [5, 24]. Both the qualitative and quantitative data from this study help shed light on a combination of individual level gaps (knowledge and skills) and systemic pressures (e.g., lack of endorsement from colleagues, time efficiency considerations, sub-optimal collaboration) that could all contribute to reducing the willingness of healthcare professionals to apply novel therapies [25]. Other studies have highlighted similar challenges to the integration of targeted therapies, especially when doing so becomes increasingly complex: for example, transitioning to these therapies often requires the development of new molecular diagnostic tests or more regular use of existing ones [26].

Communicating immunotherapy practices and how they differ from chemotherapy is an important step towards ensuring full patient understanding of their treatment plan and are engaged in the shared decision-making (SDM) process [27]. Examples of such efforts have been available online for a few years and can be used by clinicians for patient education and to engage patients in SDM process [28, 29]. Evidence from this study indicate that HCPs perceive patients as not fully understanding the particularity of immunotherapies. Lack of clarity on the roles and responsibilities of different members of the healthcare team to educate patients about their treatment options was also raised. It belongs to the health care professionals to adopt a team approach to best support patient education. As SDM in the clinical context is required by law in some US states [30], addressing the gaps highlighted here and enhancing the communication skills of HCPs in oncology can be considered essential. It is therefore imperative to develop new strategies and new tools to build and maintain a trustful patient-provider relationship [30, 31].

A potential way to address some of the issues identified here would be to establish formal communities of practice: an assembly of peers who regularly interact for the explicit purpose of deepening their knowledge and reciprocating support while increasing competence in their interest area [32, 33] in oncology settings. These have been shown to streamline interprofessional collaboration and promote a culture of partnership among the members in that type of setting [32]. Communities of practice in healthcare have been shown to contribute to better sharing of information and resources between healthcare professionals, reduced professional isolation, increase in competencies pertaining to the CoP topic, and implementation of new processes into practice [33–37]. For instance, a perceived lack of accessible data on biomarkers could account for the low skill levels participants reported in identifying those that characterize the progression of a specific type of cancer. Communities of practice in oncology settings could aid the spread and understanding of such information by providing a forum for connecting HCPs involved in this area of care with other who have various knowledge and experience levels, especially when guidelines have not fully “caught up” with options and possibilities for bedside practice [33].

As the development of new targeted therapies, including IO agents shows no sign of slowing down, it is of utmost importance to address these gaps by not only by educating HCPs to expand their knowledge of IO agents, but also by furnishing the opportunity to enhance their skills and build the necessary confidence in using pharmacodiagnostics and IO agents, as well as communicating more effectively among colleagues and with patients.

### Limitations

Participants were asked to self-report, as the study protocol did not include objective observation. This methodology can introduce different types of participant biases such as social desirability bias in both qualitative and quantitative phases [38]. To support candid reporting, both qualitative and quantitative phase participants were reminded that their data were to be anonymized. Survey scales were carefully selected to consider the tendency of physicians to overestimate their own competencies [39]. In addition, triangulation [40] and purposive sampling [41] were used to minimize self-reporting bias and selection bias (by including participants with different levels of experience, from various states and settings). Caution should still be exercised when generalizing the findings as this study included a small number of participants, where a larger study may reveal important local, and health system-specific differences. Reader should also consider response rate when interpreting reported findings. In addition, this study did not focus on one or a set of particular tumor sites, thus cancer-specific generalizations are not warranted. Further research can determine how clinical practice in pharmacodiagnostics, how IO agents may differ by cancer type and sub-types, and how specific specialist groups may experience challenges when integrating novel immunotherapies. To inform local educational activities and offerings, we recommend conducting shorter location-specific needs assessments to validate the gaps sand ensure the benefits of developing precise activities for the targeted learners.

## Conclusion

This mixed-methods study identified the main challenges and educational needs of HCPs in oncology as they increasingly integrate newly approved IO agents into practice. Educators and quality improvement experts should take these findings into consideration when developing evidence-based continuing medical education, continuing professional development, and other types of performance improvement / quality improvement interventions [42]. These findings can also help inform knowledge translation activities such as online modules and lectures designed to allow healthcare professionals to keep abreast with the latest evidence on immunotherapies. Simulation, case-based learning activities, and peer-to-peer learning opportunities (such as the development of communities of practice) can offer solutions to address the skill, confidence, and competence gaps, as well as the interprofessional collaboration challenges identified through this project.

## Supplementary Information

**Additional file 1:** **Supplementary Material A. **Sections of the interview guide, questions and probes.

**Additional file 2:** **SupplementaryMaterial B. **Selected survey questions reported in the manuscript.

## Data Availability

Due to ethical concerns (confidentiality clause of the consent form, and risk of indirect identification due to high specialisation level of the participants), raw data cannot be made openly available. Requests for further information can be sent to the corresponding author.
