# Supplementary material for: Challenges associated with the integration of immuno-oncology agents in clinical practice
Source: BMC Med Educ. 2022 Nov 12;22:781. doi: 10.1186/s12909-022-03847-0 (PMC9652913; doi:10.1186/s12909-022-03847-0)
Supplement: Supplementary file 1 — Additional file 1: Supplementary Material A. Sections of the interview guide, questions and probes. [file 12909_2022_3847_MOESM1_ESM.docx]

**Supplementary Material A: Sections of the interview guide,
questions and probes**

| **Interview sections** | **Examples of open questions** | **Examples of probes** |
| --- | --- | --- |
| Understanding practice setting, roles, and tasks of interviewee | **ONCO/NP/PA/PHARM/Pulm/RH:** What are your main roles and tasks in the care of cancer patients receiving immuno-therapy? | - Why? - What do you find challenging about that? - Can you talk to me more about…? - Can you give me an example of…? - Can you give me an example of…? - How does this affect your work? |
| Challenges faced in care of patients treated with immunotherapy | What are the two greatest challenges that you face in relation to the care of cancer patients treated with immunotherapy? |  |
| Challenges faced in selection of pharmacodiagnostics | What is your main source of pharmacodiagnostics-related information? |  |
|  | **PA/NP/Pulm**: In your practice, is genomic testing (biomarker testing) used to predict patient response to treatment? |  |
| Challenges faced in considering efficacy and safety of new I-O agents for specific patient profiles | **ONCO:** What factors do you consider when deciding whether to treat a patient with a new I-O agent |  |
|  | **ER**: If a patient is receiving I-O treatment, how does this impact your decision-making about when or if to discharge them from the hospital? |  |
|  | **ONCO/RH/ER:** Do you use a patient monitoring system (e.g., electronic medical records or EMR system) to help recognise toxicities and other irAEs? |  |
| Challenges faced in communication with patients about treatment preferences. | **ALL:** How would you describe your experiences communicating with patients who are being treated with immuno-oncology agents? |  |

Legend:

ONCO: Oncologist

RH: Rheumatologist

ER: Emergency department physician

Pulm: Pulmonologist

PATH: Pathologist

IR: Interventional radiologist

PHARM: Clinical pharmacist

PA: Physician assistant

NP: Nurse practitioner
