# Supplementary material for: Challenges associated with the integration of immuno-oncology agents in clinical practice
Source: BMC Med Educ. 2022 Nov 12;22:781. doi: 10.1186/s12909-022-03847-0 (PMC9652913; doi:10.1186/s12909-022-03847-0)
Supplement: Supplementary file 2 — Additional file 2: SupplementaryMaterial B. Selected survey questions reported in the manuscript. [file 12909_2022_3847_MOESM2_ESM.docx]

**Challenges in Immunotherapies**

**Supplementary Material B: Selected survey questions reported in the manuscript**

Participants were shown the following text prior to participation: “You do not have to answer any questions that you do not want to answer, and you may stop participating in this screening process at any time. Please note that participant space may be limited for specific categories of participants, to ensure that the final sample is representative of American providers. You may only participate once.”

1. **In which state do you practice / work?**
2. **What is your primary profession?**
3. Physician
4. Nurse Practitioner
5. Physician Assistant
6. Clinical Pharmacist
7. Other (please specify) (disqualify)
8. **What is your primary specialty?**
9. Oncology
10. Interventional radiology
11. Emergency medicine
12. Rheumatology
13. Pulmonology
14. Pathology
15. Other (please specify) (disqualify)

**3b. Which of the following procedures do you perform? (select all that apply)**

1. Image-guided biopsies (mandatory)
2. Embolization procedures
3. Ablation procedures
4. **(if NP or PA) What is your primary specialty?**
5. Primary Care (disqualify)
6. Internal medicine: Oncology
7. Other internal medicine sub-specialties (disqualify)
8. Surgery: Oncology
9. Other surgery sub-specialties (disqualify)
10. Pediatric subspecialties (disqualify)
11. Radiation oncology
12. Emergency medicine (disqualify)
13. Other (please specify) (disqualify*)*
14. **What BEST describes your main work setting?**
    1. Solo practice
    2. Single-specialty physician group practice
    3. Multi-specialty physician group practice
    4. Community clinic
    5. Government medicine (such as Veterans Affairs)
    6. Academic or academic-affiliated hospital or medical center
    7. Hospital (community-based - non-affiliated)
    8. Other (please specify)
15. **Please indicate how strongly you agree or disagree with each statement below.**

|  | **Strongly disagree** | **Disagree** | **Neutral** | **Agree** | **Strongly agree** |
| --- | --- | --- | --- | --- | --- |
| In my setting, electronic medical records are not always up to date regarding new treatments | 1 | 2 | 3 | 4 | 5 |
| Despite the volume of new agents available, very few constitute real innovations | 1 | 2 | 3 | 4 | 5 |
| Current guidelines do not reflect current treatment landscape | 1 | 2 | 3 | 4 | 5 |
| I prefer prescribing treatments I am familiar with, unless the risk-reward is important | 1 | 2 | 3 | 4 | 5 |
| I expect other health care professionals in charge of completing the biopsy to know exactly how much tissue is required | 1 | 2 | 3 | 4 | 5 |

1. **Reflecting on your practice, for each item listed below, please rate your current level of knowledge, considering how relevant that knowledge is to your current role.**

|  | **No knowledge at all** | **Basic knowledge** | **Intermediate knowledge** | **Advanced knowledge** | **Expert knowledge** |
| --- | --- | --- | --- | --- | --- |
| Safety profile of Avelumab | 1 | 2 | 3 | 4 | 5 |
| Safety profile of Alemtuzumab | 1 | 2 | 3 | 4 | 5 |
| Safety profile of Belimumab | 1 | 2 | 3 | 4 | 5 |
| Best practices for treatment of patients with lupus using immuno-oncology agents | 1 | 2 | 3 | 4 | 5 |
| Best practices for treatment of patients with rheumatoid arthritis or ankylosing spondylitis using immuno-oncology agents | 1 | 2 | 3 | 4 | 5 |
| Best practices for treatment of patients with psoriasis and/or psoriatic arthritis using immuno-oncology agents | 1 | 2 | 3 | 4 | 5 |
| Sources of information that can improve patients’ understanding of immuno-oncology agents | 1 | 2 | 3 | 4 | 5 |
| Sources of information that can mislead patients’ understanding of immuno-oncology agents | 1 | 2 | 3 | 4 | 5 |

1. **Reflecting on your practice, for each item listed below, please rate your current level of knowledge, considering how relevant that knowledge is to your current role.**

|  | **No skill at all** | **Basic skill** | **Intermediate skill** | **Advanced skill** | **Expert skill** |
| --- | --- | --- | --- | --- | --- |
| Explaining to patients the difference between immuno-oncology agents and chemotherapy | 1 | 2 | 3 | 4 | 5 |
| Weighing the risks and benefits of treating patients with lupus with immuno-oncology agents | 1 | 2 | 3 | 4 | 5 |
| Identifying viable treatment options based on pharmacodiagnostic test reports | 1 | 2 | 3 | 4 | 5 |
| Identifying markers that will characterize the progression of a specific type of cancer | 1 | 2 | 3 | 4 | 5 |

1. **Reflecting on your practice, for each item listed below, please rate your current level of confidence, considering how relevant that knowledge is to your current role.**

|  | **No confidence at all** | **Slightly confident** | **Somewhat confident** | **Confident** | **Very Confident** |
| --- | --- | --- | --- | --- | --- |
| Setting realistic expectations with my patients regarding the impact of immuno-therapy on their cancer | 1 | 2 | 3 | 4 | 5 |
| Explaining to my patient their ineligibility for immuno-therapy, due to health status | 1 | 2 | 3 | 4 | 5 |
| Explaining to my patient why they are not responding positively to a given immuno-therapy agent | 1 | 2 | 3 | 4 | 5 |
